# Public Health and Sanitation

**Published:** 1897-08

**Authors:** 


### Public Health and Sanitation.

The Women's Health Protective Association of Brooklyn recently held its annual assembly. The object of the association is to coöperate with the health department in securing improved sanitation. Some of the most prominent women in Brooklyn are members.

The following interesting facts were given in the secretary's report : The seventh year of the Women's Health Protective Association closes with this meeting. Progress has been made all along the lines laid down in previous years. In the line of practical work the reform of the ash barrel and the garbage receptacles is still our aim. It is still our endeavor to gain the coöperation of our sister-citizens in keeping the barrels in neat condition, in having ashes and rubbish separated and all receptacles for garbage whole and durable. The crusade against the objectionable habit of spitting has been carried on. Spitting in public conveyances is now punishable as a misdemeanor in New York, Boston, Philadelphia and San Francisco, while in Philadelphia signs forbidding it are also placed on lamp posts and trolley poles.

